# Articulating Teeth on Rubber Work

**Published:** 1862-04

**Authors:** 


### Editorial.

### ARTICULATING TEETH ON RUBBER WORK.

ONE great difficulty in constructing rubber work is the derangement of the teeth in regard to articulation. However well the teeth may be articulated according to the ordinary method, when the work is finished, as it is commonly done, the articulation will be found more or less imperfect. This occurs by the model being in one part of the flask, and the teeth in the other, when the rubber is packed; the two parts then being closed, if they fail to come together as perfectly as when they are filled with plaster, by just that much will the teeth be out of the intended articulation; and it is almost impossible to bring the parts of the flask entirely together, owing to the intervening rubber.

It is true grooves in the plaster should be made for this, but it is difficult to confine all the surplus to these channels. A plan has been suggested and adopted by Dr. L. A. Hendricks, by which this difficulty is wholly obviated; this is accomplished by arranging so that the teeth shall remain in their exact relative position with the model, during the packing of the rubber, and the compression.

This is done by building up a thick wall of plaster, all round and upon the outer surfaces of the teeth, as they stand upon the model, after the wax is put on and trimmed properly. This plaster wall extends from the crowns of the teeth, to the border of the model part of the flask. This is trimmed and varnished, and then the upper portion of the flask is added and filled, as usual; then, when the flask is separated, the teeth remain upon the model, instead of coming off with the upper part of the flask.

The wax and pattern plate is then removed, and the rubber packed as usual, care being taken to pack it well under the teeth, using small pieces at first, and to leave an excess in that vicinity, that it may be pressed under sufficient to form the rim; the sections of the flask are then closed. In this way there is no liability of breaking the gum from the teeth or blocks, however thin it may be. Fracture of the gum very frequently occurs, by the old method.

It is worthy of remark here, that the wax which forms the mould for the rim can not be removed with instruments, but it will be absorbed by the plaster when it is heated, preparatory to packing; or what is perhaps better, put the piece containing the teeth and model, after separation, into boiling water; the wax will thus become melted and pass out on the water. In putting rubber rims on gold plates, with rubber attachment, if the teeth fit closely upon the plate, sufficient rubber may not be forced through, to form the rim complete; if there is any doubt in regard to that, after the piece has been packed and pressed together, cut through the plaster from the under side, down to the rim mould, and if it be found deficient, pack in sufficient rubber to make it complete, then cover up with plaster. This can not be done with the old fashioned flasks, but only with those made with Hays' machine, so far as we now know. T.

